# Micro‐Scale Ice Shoveling Effect Induced by Magnetic‐Responsive Microfins

**DOI:** 10.1002/advs.202408594

**Published:** 2024-10-23

**Authors:** Yiyi Chen, Ming Liu, Lijing Zhou, Jian Deng, Xianghui Hou, Xuerui Mao

**Affiliations:** ^1^ Advanced Research Institute of Multi‐Disciplinary Sciences Beijing Institute of Technology Beijing 100081 China; ^2^ School of Mechanical Engineering Beijing Institute of Petrochemical Technology Beijing 102617 China; ^3^ School of Aerospace Engineering Beijing Institute of Technology Beijing 100081 China; ^4^ State Key Laboratory of Fluid Power and Mechatronic Systems Department of Mechanics Zhejiang University Hangzhou 310027 China; ^5^ State Key Laboratory of Solidification Processing Shaanxi Key Laboratory of Fiber Reinforced Light Composite Materials Northwestern Polytechnical University Xi'an 710072 China; ^6^ Beijing Institute of Technology Zhuhai Beijing Institute of Technology (BIT) Zhuhai 519088 China; ^7^ State Key Laboratory of Explosion Science and Safety Protection Beijing 100081 China

**Keywords:** ice shoveling effect, inhomogeneous deformation, magnetic‐responsive surface

## Abstract

Icing is ubiquitous in nature and engineering applications, and imposes threats to road and air transportations, wind energy infrastructures, etc. However, current active de‐icing solutions, especially the most popular one, i.e., heating, suffer from high energy consumption whilst passive methods are often ineffective at high‐speed, long‐term, or large‐particle conditions. Herein, a promising strategy adopting magnetic‐responsive microfins (MRS) featuring reversible deformations is developed for de‐icing. A novel micro‐scale ice shoveling effect induced by the localized destruction of the ice adhesion interface owing to the inhomogeneous deformation is demonstrated, and its dependence on the ice particle size and temperature is investigated. An analytical model is proposed to describe the mechanism of this effect, showing a linear relation between the position of the magnet and the induced force agreeing well with experiments, leading to a system straightforward to predict and control. Specifically, the de‐icing capacity of the surface becomes prominent when small‐scale ice particles merge to large ones, providing a promising solution for applications on aircraft, wind turbines, etc., as the first of its kind to remove large particles under high‐speed conditions effectively.

## Graphical abstract

This study develops a new de‐icing solution by constructing a magneticdriven moveable surface. A novel micro‐scale ice shoveling effect is induced by the localized destruction of the ice adhesion interface owing to the inhomogeneous surface deformation is demonstrated. This in‐situ de‐icing method without melting step provides an idea for further developments of efficient and reliable deicing solutions.

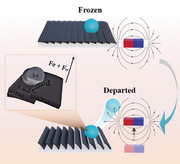

## 1 Introduction

Icing as a common natural process has been extensively exploited in sports, food storage, sculpture, etc., to benefit human life, but it is also notorious in leading to serious safety concerns and economy losses in many other scenarios, e.g. aviation, power transmission, and wind energy.^[^ ^1^, ^2^, ^3^ ^]^ Ice accretion on the aircraft surface is caused by impacting and freezing of water droplets, which degrade the aerodynamic performance and endangers flight safety by reducing lift and increasing drag.^[^ ^4^ ^]^ Similarly, surface icing on wind turbines or transmission cables leads to power loss and damage of devices.^[^ ^5^ ^]^

Due to these negative impacts of icing, extensive investigations on efficient de‐icing methods, including active, passive or hybrid ones, have been reported. Active methods mainly refer to heating,^[^ ^6^ ^]^ mechanical vibration^[^ ^4^, ^5^, ^6^, ^7^ ^]^ and chemical fluid,^[^ ^8^ ^]^ where the first is generally considered as the most effective one. However, heating requires complex energy supply systems and long response time and is also energy inefficient.^[^ ^9^ ^]^ More seriously, re‐freezing phenomena widely exist in heating strategies as the melted droplets tend to stick to the cold surface and act as the base of the secondary icing. Such re‐freezing can be alleviated by increasing the power supply or the de‐icing area, which inevitably exaggerates the energy consumption.

Passive methods mainly utilize surface multi‐level structures^[^ ^10^, ^11^, ^12^, ^13^, ^14^, ^15^, ^16^ ^]^ or anti‐icing coating materials^[^ ^17^, ^18^, ^19^, ^20^, ^21^ ^]^ to minimize the ice formation or reduce the surface adhesion.^[^ ^14^, ^22^, ^23^, ^24^ ^]^ Although superhydrophobic surfaces exhibit icephobic features by reducing the contact area and the ice adhesion strength, the coatings or designed structures are subject to losses due to poor mechanical strength, leading to concerns on effectiveness, environment, and duration, etc.^[^ ^25^, ^26^, ^27^, ^28^ ^]^

There have been growing efforts devoted to new strategies for anti/de‐icing, such as slippery liquid‐infused porous surface (SLIPS) and liquid‐like slippery surface (LLSS), both of which exploit slippery effects to reduce ice adhesion strength and enhance the anti/de‐icing performance. Different from SLIPS relying on lubricating liquid on the solid surface,^[^ ^3^, ^29^, ^30^ ^]^ LLSS works by installing flexible molecular brushes such as Polydimethylsiloxane (PDMS) onto the surface. These brushes lead to a solid/liquid composite interface between the ice and the surface, more stable than air pockets on superhydrophobic surfaces and surfaces without introducing complex structures. In addition to reducing the ice adhesion strength, the effect of slippery also efficiently delays ice formation by prohibiting the heterogeneous nucleation of water.^[^ ^29^, ^30^, ^31^ ^]^

Inspired by SLIPS and LLSS, which activate the slippery effect by exploiting moveable and mobile behaviors to overcome the limitation of superhydrophobic surfaces, we develop a new magnetic‐responsive anti/de‐icing strategy resorting to the micro‐scale ice shoveling effect. This approach exhibits both active and passive effects and is expected to be more efficient than the pure passive SLIPS and LLSS partly because it destructs the ice‐substrate interface locally rather than globally. Magnetic particle‐based structures are selected as they feature high shape recovery and fast deformation rate because of their intrinsic prompt response to an external magnetic field.^[^ ^32^, ^33^, ^34^ ^]^ Such structrures have been studied for anti‐icing, e.g. switching adhesion, wetting and rebound of a droplet, but their deicing capacities based on the structure deformation have not been actively explored. In the present novel strategy, magnetic‐responsive surfaces (MRS) with multi‐level structures exhibit reversible structural deformation and meanwhile maintain hydrophobic and ice‐phobic features. The hydrophobic multi‐level structure presents high flexibility and chemical and thermal stability, which are beneficial to lift the durability performance. In the de‐icing process, the in‐situ structural deformation produces a tangential stress, shoveling the ice particle from the surface even at temperatures as low as −40 °C. We further establish an analytical model for the dependence of the ice shoveling effect on the ice particle size and the environmental temperature. Overall, the proposed in‐situ deicing solution without energy‐intensive heating offers an idea for further developments of efficient and reliable deicing solutions for aircraft, wind turbines and power transmission.

## 2 Results and Discussions

### 2.1 MRS Design and Fabrication

#### 2.1.1 Fabrication of the MRS

Hydroxy iron powders (HIPs) with an average size 5–10 µm were purchased from Shanghai Naiou Nano Technology Co., Ltd,. Polydimethylsiloxane (PDMS) and curing agent (Sylgard 184) were acquired from Dow Corning, America. The photopolymer precursor (GR‐20) used in 3D printing is provided by BMF Material Technology Inc., China. 1H,1H,2H,2H‐Perfluorodecyltrichlorosilane used as the fluoroalkylsilane was from Aladdin Co., China.

**Figure 1:**
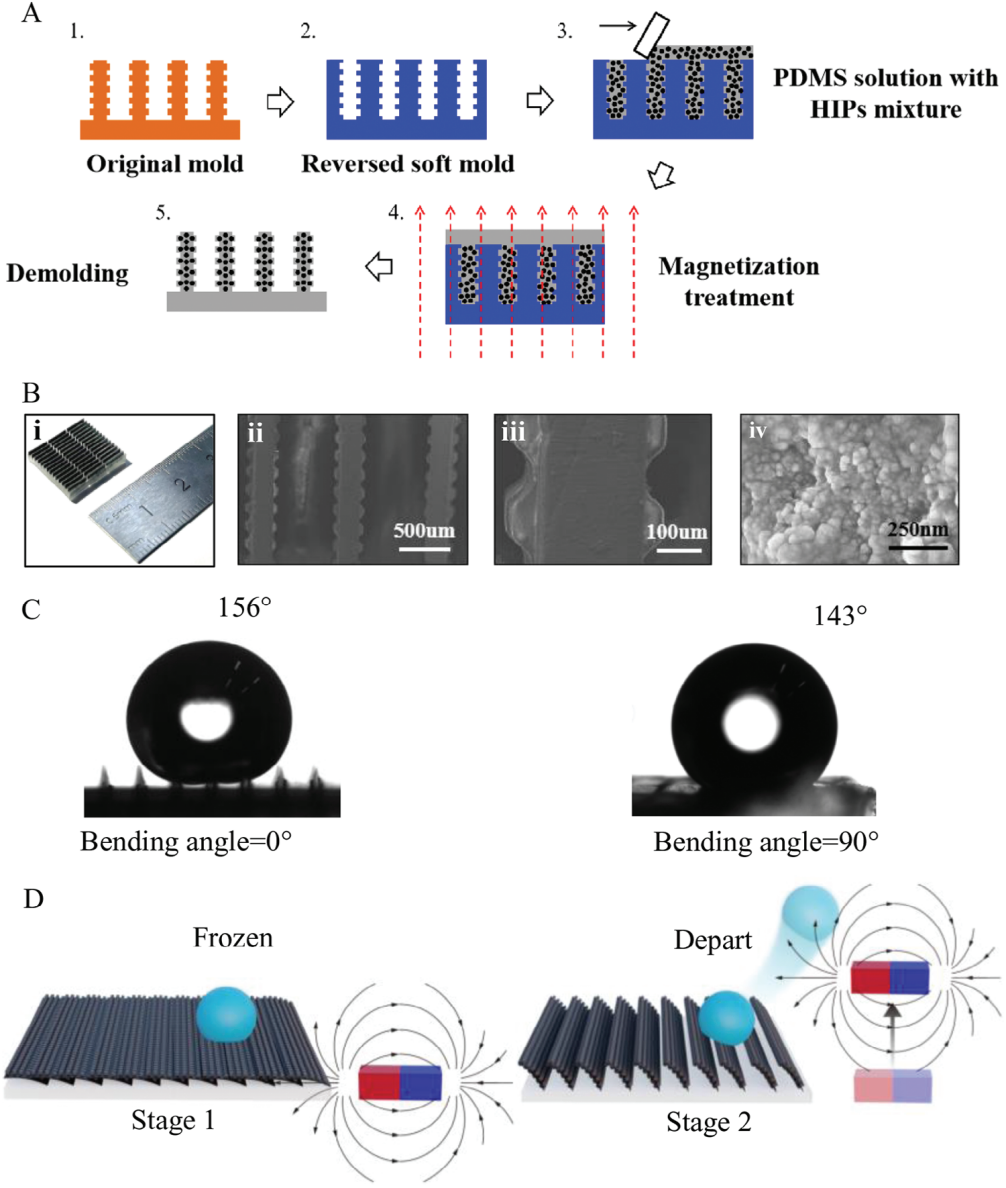
A) Main steps to fabricate the magnetic‐responsive surfaces (MRS), B) morphology of the surface: (i) full scale of MRS, (ii) SEM image of microfins, (iii) SEM image of micro‐pillar and (iv) SEM image of nano particles, C) droplet contact angle of the MRS with different tilt bending angle, and D) schematic view of the process to remove ice particles from the surface exposed to an external magnetic field.

These materials enable the MRS to be fabricated following steps illustrated in **Figure** **1**A. In step 1, the original mold was designed and fabricated using a 3D printer (nanoArch S140, BMF Material Technology Inc., China), which was cleaned for 5 min by ultrasonication in ethyl alcohol to remove the residual photopolymer solution. To avoid the sticking of polymer in the next step, the original mold was exposed to fluoroalkylsilane for 10 h in vacuum. In step 2, the PDMS mixture with precursor and curing agent mixed with a 10:1 ratio was poured on the original mold and degassed. After curing for 2 h at 80 °C, the soft mold with reverse microstructures was obtained. The silanisation treatment was also applied to the soft mold. In steps 3–4, the PDMS solution with a 10:1 ratio of the precursor to curing agent was mixed with HIPs (60 wt%), the homogeneous suspension solution was poured on the coated soft mold and the residual precursor layer was scraped away by a glass slide. Then, the pure solution of PDMS with a 10:1 ratio was poured on the surface of the soft mold. After the magnetization treatment in a uniform 0.25T magnetic field for 5 min and cured for 30 mins at 80 °C, crosslinked microstructures with ferromagnetic particles arranged in chains were formed in the soft mold. In step 5, the MRS was obtained after demolding. To enhance the water repellence performance, the nanoscale silica particles were sprayed onto the surface microstructures by a retail sprayer (Soft 99).

The morphology of the MRS with multi‐level structures was measured by a field‐emission scanning electron microscope (S4800, Tescan, China). The magnetic‐responsive fin is black because of the filling of magnetic particles and illustrates a clear structural periodicity, as shown in Figure 1B(i). Periodic micro‐pillars covering the left and right lateral surfaces of the microfins are highlighted in Figure 1B(ii),(iii). Each pillar has a dimension of 90 µm × 90 µm  × 30 µm. Figure 1B(iv) shows the surface coated with silica nanoparticles with an average size of 100 nm to enhance the surface hydrophobicity and icephobicity. The length, width, height and pitch of each fin are designed as 5, 0.15, 1 and 0.8 mm, respectively.

In addition, the surface wettability was measured by the contact angle analyzer (OCA25, Dataphysics, Germany), during which the used droplet volume was 5 µl. Figure 1C shows images of sessile millimetric water droplets on the surface under two tilt bending angles (20 °C, 25% in humidity). To verify the uniformity of the MRS, more than 5 tests were carried out at different locations of the surface, and the average contact angle of the surface at 0° and 90° tilt bending angle was 154.1° and 143.6°, respectively, with the maximum error below 2%. It is noted that the MRS presents an excellent hydrophobic property regardless of the tilt bending angle.

In the test of the uniform magnetic field, two permanent NdFeB magnets with the same dimension of 100 mm × 50 mm × 10 mm were adopted. The mechanical deformation of the surface microstructure was driven by another two permanent magnets with the dimensions of 50 mm × 15 mm × 10 mm. Figure 1D illustrates the main process to remove ice particles from the surface exposed to external magnetic fields. In stage 1, fins can be treated as fully tilted at a high magnetic flux, and the water droplet is frozen on the surface. In stage 2, under a vertical motion of the magnetic field, the interface between ice and substrate can be destroyed by the bending of microfins, leading to ice detachment from the surface.

#### 2.1.2 Icephobicity and Deformation Behaviours of the MRS

**Figure 2:**
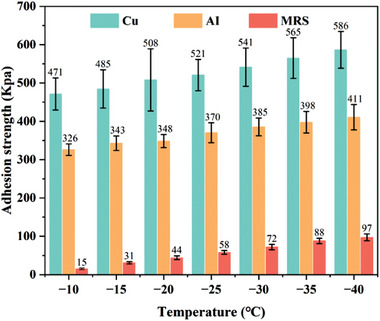
Adhesion stress between ice and the MRS and metal plates under different temperature conditions.

A critical parameter evaluating the surface icephobicity is the adhesion strength between the surface and ice and also quantifies the difficulty of de‐icing. The measurement of the shear ice adhesion force is illustrated in Figure S2 (Supporting Information). A hollow column with distilled water filled freezes and sticks to the cold test surface. Then a force transducer records the critical force to detach the ice column from the test surface using the lateral pushing method. **Figure** **2** shows the measured ice‐adhesion results at different temperature of the MRS with the bending angle of 90° and reference surfaces. The mean value was calculated from at least three individual measurements and the bars represent the standard deviation, which may stem from material uncertainty, fluctuations of environmental conditions, etc. The adhesion strength of the MRS ranges from 15Kpa to 97KPa as the temperature decreases from −10 to −40 °C, much lower than the polished copper and aluminum surface and can be defined as a low adhesion surface (τ < 100 KPa). It is worth mentioning that the passive reduction of the adhesion strength is not the main focus of this work.

**Figure 3:**
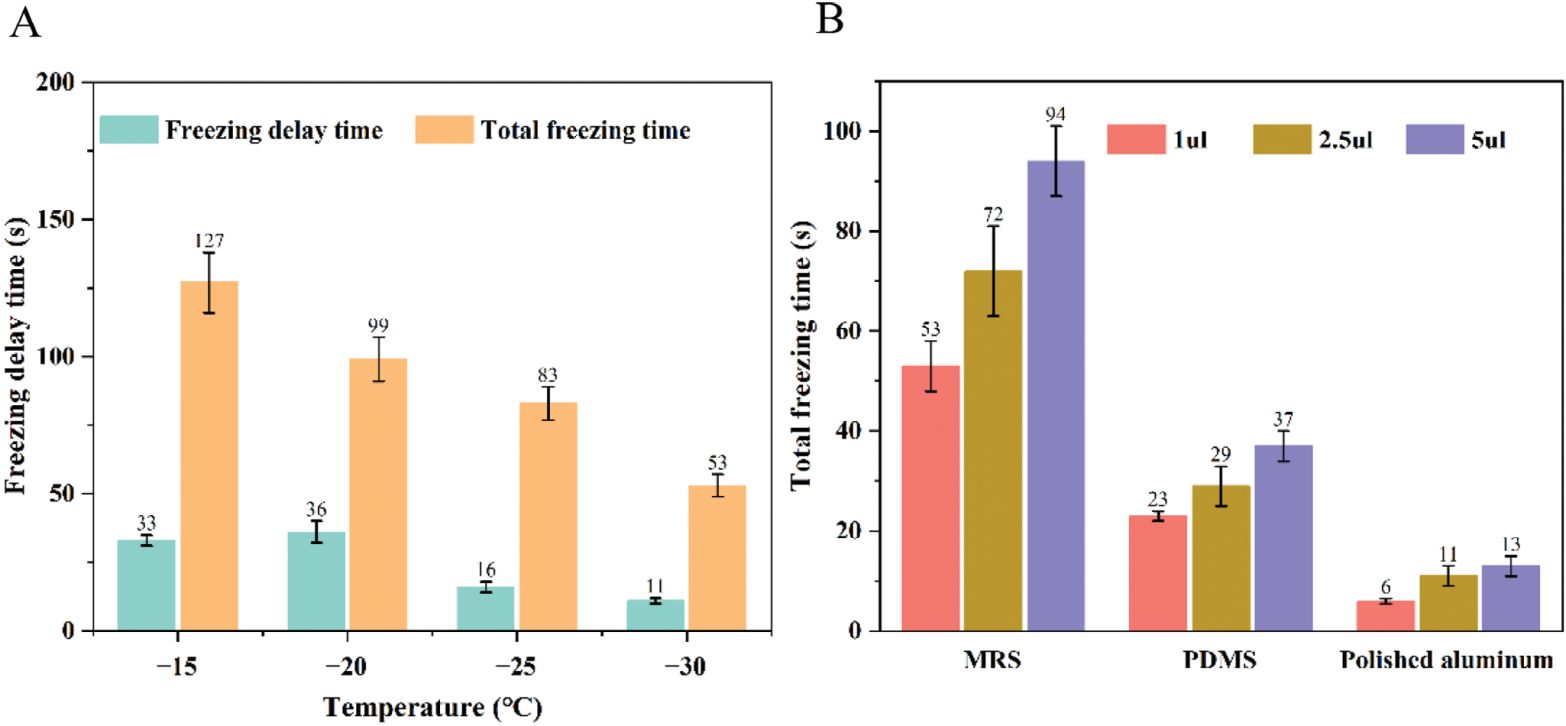
A) Freezing delay time and total freezing time of the water droplet with the size of 1 µL on the MRS at different temperatures. B) Total freezing time of different sizes of water droplets on the MRS, PDMS and polished aluminum surfaces at −30 °C.

The freezing delay and total freezing time of water droplets with the size of 1 µL and surface temperature of −15 and −30 °C on the MRS are illustrated in **Figure** **3**A. As expected, the time is extended at a higher temperature due to the slower nucleation rate. Figure 3B shows the comparison of total freezing time of water droplets with size from 1 to 5 uL on the MRS and other referenced surfaces at surface temperature −30 °C. Based on Fourier's law of heat conduction, the thermal resistance of heat transfer from the surface to the ice particle is proportionate to $\frac{H}{A}$, where *H* is the height of the ice particle. Due to the hydrophobic property of the surface, the ratio of height over the contact area *A* increases with the droplet size, leading to a higher thermal resistance and longer total freezing time as shown in Figure 3B. In addition, the total freezing time of PDMS and aluminum surfaces with the same thickness was measured. Compared with these two referenced surfaces, the freezing time of the MRS is extended significantly due to the high thermal resistance of air pocket trapped around the interface.

In the following, the reversible bending deformation of microfins in response to a uniform external magnetic field with flux density 0.6T is tested. The field is generated by two permanent magnets placed in parallel and with opposite poles and the tested surface is placed between the magnets. The angle between the surface and the field can be adjusted by rotating the surface.

**Figure 4:**
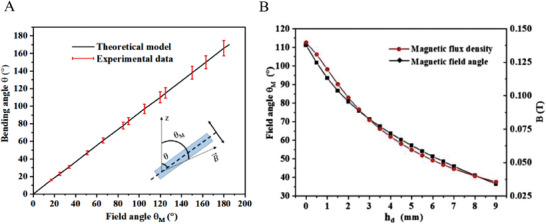
A) The variation of the bending angle θ with respect to the magnetic field angle θ_*M*_, predicted by the experiment and theoretical model. B) The effect of magnet height *h* on the magnetic flux density and the magnetic field angle θ_*M*_ at the location of the ice droplet.

The magnetic field induces a torque and correspondingly the deformation of the microfins and the reaction moment due to its elasticity. The deformation can be quantified by the bending angle θ, which varies with respect to the magnetic field angle θ_*M*_. Both angles are measured from the vertical axis as shown in **Figure** **4**A. Here the microfin is subject to the elastic force *F_M_* and the magnetic force *F_e_* ^[^ ^34^ ^]^

(1) $$F_{M}=VPBsin\left(\theta_{M}-\theta \right)$$

(2) $$F_{e}=\theta /k$$

where *k*, *V*, *P* and *B* are constants related to the material of the microfin, the volume of a single microfin, the saturating magnetization intensity and the magnetic flux density of the magnetic field, respectively. The distribution of the magnetic field and subsequently *B* is computed in Comsol Multiphysics software. The bending angle can be obtained by balancing these two forces:

(3) $$\theta =k\;VPB\sin\left(\theta_{M}-\theta \right)$$

The experimental data in Figure 4A shows a proportional relation, i.e., θ_*M*_ =  αθ, with α ≈ 1.^[^ ^35^ ^]^ Retaining the linear part of the Taylor expanded form of sin (θ_*M*_ − θ) and substituting into (3), there is:

(4) $$\theta =\frac{k\;VPB}{1+k\;VPB}\;\theta_{M}$$

The theoretically predicted bending angle from equation (4) is in good agreement with experimental measurements, as shown in Figure 4A,B presents the variation of the magnetic flux density *B* and the magnetic field angle θ_*M*_ at the location of the ice droplet with respect to the magnet height *h* measured from the horizon of the MRS. Only positive values of the height are plotted since the negative part is symmetric. As expected, when lifting the magnet, its impact reduces, including the reduction of the magnetic flux density, the magnetic field angle θ_*M*_ and the deformation angle θ.

#### 2.1.3 Micro‐Scale Ice Shoveling Effect of the MRS

**Figure 5:**
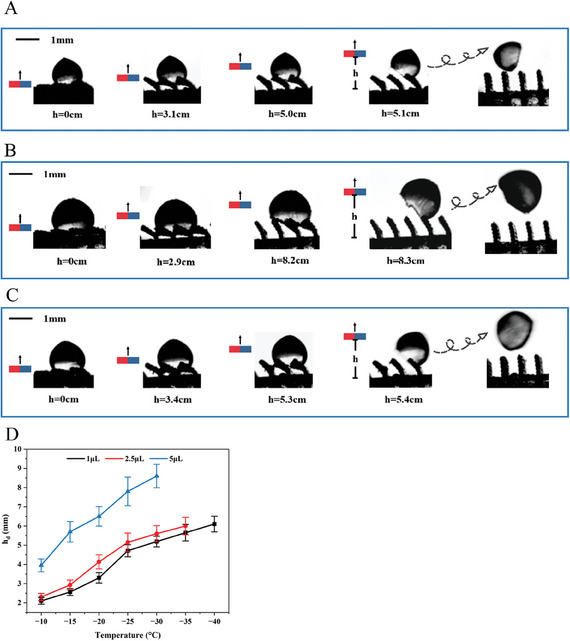
De‐icing process captured by a high‐speed camera at surface temperature −30 °C and size of droplet. A) 1, B) 2.5, and C) 5. D) Critical height *h_d_* to remove the particle at different particle sizes and surface temperatures.

The removal of ice particles from the MRS is evaluated by using de‐icing experiment test rig shown in Figure S3 (Supporting Information). When the magnet block is directly placed on the lateral of the surface at *h* = 0, the field intensity in the region where the location of the ice particle is 0.14T, which is computed using Comsol Multiphysics solftware (see Figure 4B). At this moment, the fins can be treated as fully tilted (see stage 1 in Figure 1D). Then deionized sessile water droplets are attached on the lateral of the fins with size 1, 2.5 and 5 µL and surface temperature −30 °C, as shown in **Figure** **5**A‐C, respectively. Under a vertical motion of the magnet, a pushing force is gradually produced and increases with the deformation of the microfins. Theoretically, once the shear of the force increases to the value of the ice adhesion strength, the interface between ice and substrate can be destroyed. At *h* = 5.1 cm, the ice particle with size 1 µL is totally shoveled and detached from the surface of the left fin and this critical height, denoted as *h_d_* in the following, increases with the size of the particle.

Figure 5D shows the effect of temperature on *h_d_* at different particle volumes. For all three cases, *h_d_* is larger at lower temperature due to the increase of ice adhesion force. At particle volume 1 µL, ice detachment is observed until −40 °C, lower than which no detachment can be obtained by lifting the magnet in the present setup. For particles with size 2.5 and 5 µL, this critical temperature rises to −35 and −30 °C, respectively. Moreover, larger particle requires a larger *h_d_* to induce the detachment.

#### 2.1.4 Mechanism of the Ice Shoveling Effect

**Figure 6:**
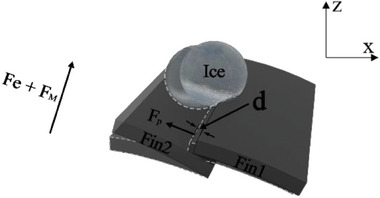
Schematic plot of the ice removal model.

A theoretical model is established to address the mechanism of the ice shoveling effect induced by the MRS. **Figure** **6** illustrates the de‐icing process and the forces acting on the ice particle covering two fins, e.g. with size 1 and 2.5 µL shown in Figure 5, respectively. When lifting the magnet, the fins start to deform driven by the elastic force *F_M_* and the magnetic force *F_e_*, and a relative motion between fin 1 and fin 2 is generated along the fin's direction. This motion is terminated by the particle attached to the fins. Consequently, fin 1 is squeezed until the pushing force *F_p_* generated by fin 1 is larger than the adhesion force *F_n_*. The magnitude of the relative motion equals the change of the distance between the tips of the two fins along the fin direction, which is denoted as *d* and can be computed as

(5) $$d=S_{fin}\;\left[\sin\left(\theta_{0}\right)-\sin\left(\theta \right)\right]$$

where *S_fin_* is the spacing between the fins and θ_0_is the initial bending angle of the fin determined by the de‐icing experiment. Then *F_p_* can be obtained from^[^ ^36^ ^]^

(6) $$F_{p}=E\frac{d}{A_{2}L_{Fin}}A_{1}$$

where *E* is the Young's modulus of the fin, *L_Fin_* is the length of the fin. *A*_1_ and *A*_2_ are the contact areas between the particle and fin 1 and fin 2, respectively. The deductions of *A*_1_ and *A*_2_ are given in Supplementary Information. By substituting Equation (4) and (5) into Equation (6), we obtain

(7) $$F_{p}=\frac{EA_{1}S_{fin}\left[\sin\left(\theta_{0}\right)-\sin\left(\frac{k\;VPB}{1+k\;VPB}\theta_{M}\right)\right]}{A_{2}L_{Fin}}$$

where *B* and θ_*M*_ are determined by *h_d_* (see Figure 4B).

For large particles covering three fins, e.g. with size 5 µL, given that the detachments from the last two fins take place at the same time as shown in Figure 5C, it is reasonable to suppose that the detachments occur when *F_p_* generated by the first fin is larger than the sum of the adhesion force on the last two fins. In this situation, *A*_2_ is the sum of the contact area on the last two fins, and *A*_1_ is that on the top of the first fin.

When the height of the magnet increases, θ_*M*_ reduces (see Figure 6A), so does θ (see Equation 4), and subsequently from Equation 5, *d* increases, leading to the increase of *F_p_* (see Equation 6). When *F_p_* is larger than *F_n_*, the pushing force overcomes the ice adhesion strength, and detachment occurs.

**Figure 7:**
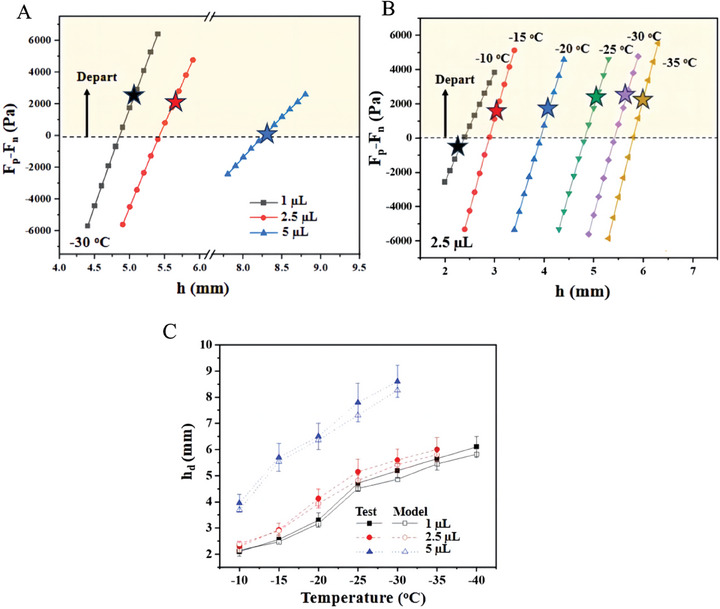
Comparison of experimental results with the analytical model. A) Ice particle size effects on the pushing force at −30 °C. B) Temperature effects on the pushing force generated by ice particles with the size of 2.5 µL. C) Particle size and temperature effects on the critical height.

Following Equation (7), the value of *F_p_* − *F_n_* increases from negative to positive with the rising of the magnet height, as shown in **Figure** 7 with different particle volumes and temperatures, respectively. The dashed line represents *F_p_* −  *F_n_* =  0, where the particle detachment is supposed to occur. The star symbols represent the experimentally measured critical value of the height *h_d_* at which detachment occurs. The vertical distance between the star and the dashed line quantifies the accuracy of the theoretical model (Equation 7).

The straight lines of the analytical solution in Figure 7A,B imply that there is a linear relation between *h*_d_ and *F_p_* regardless of the particle size and the temperature. From Equation (7) only $\sin(\frac{k\;VPB}{1+k\;VPB}\theta_{M})$ depends on *h_d_*. Performing a data fitting in Figure 4B, there is $\frac{k\;VPB}{1+k\;VPB}\theta_{M}\approx \arcsin\left(-0.076\;h_{d}+0.91\right)$ substituting this to Equation (7), there is

(8) $$F_{p}\approx \frac{0.076EA_{1}S_{fin}}{A_{2}L_{Fin}}h_{d}$$

illustrating a linear dependence of *F_p_* on *h*_d_, agreeing with Figure 7A,B and indicating that the system is straightforward to predict and control.

Figure 7C summarizes the critical height *h_d_* at different particle volumes and temperatures. Both predicted and experimentally measured values increase with reduced temperature at all three particle volumes, which can be attributed to the increase of ice adhesion force. The average deviation between the model and the measurement is 4.2%, demonstrating the accuracy of the established model.

## 3 Discussion

In this work, we developed a new de‐icing solution by constructing a magnetic‐driven moveable surface and observed a novel micro‐scale ice shoveling effect, taking advantage of the inhomogeneous forces acting on the ice‐substrate interfaces. The ice particle detachment is triggered by the fast response and quick deformation of hydrophobic magnetic microfins without heating and consequently avoiding the re‐freezing problem. It is demonstrated that the ice and substrate interface can be destroyed once the shear force induced by the structure deformation increases to the value of the ice adhesion strength. Specifically, the maximum de‐icing capacity of the surface becomes prominent when small‐scale ice particles merge to large ones up to 5 µL, which offers the potential for a wide range of applications on de‐icing. Further, an analytical model to analyze the mechanism of de‐icing is established, illustrating that the surface induced force to detach the particle is linear with respect to the controlling parameters related with the magnet fields, leading to a system easy to predict and control.

## 4 Experimental Section

### Material

Hydroxy iron powders (HIPs) with an average size of 5–10 µm were purchased from Shanghai Naiou Nano Technology Co., Ltd., Polydimethylsiloxane (PDMS) and curing agent (Sylgard 184) were acquired from Dow Corning, America. The photopolymer precursor (GR‐20) used in 3D printing is provided by BMF Material Technology Inc., China. 1H,1H,2H,2H‐Perfluorodecyltrichlorosilane used as the fluoroalkylsilane was from Aladdin Co., China. These materials enable the MRS to be fabricated following steps illustrated in Figure 1A.

### Ice Adhesion Strength Measurement

Measurement of the ice shear adhesion strength is conducted on a cooling plate with temperature from −40 °C to −10 °C, as shown in Figure S2 (Supporting Information). A hollow column with 9 mm in inner length and width is put on the test surface and then 10 ml distilled water is filled for 1 hour to form an ice column sticking to the test surface. A force transducer is used to record the critical force to detach the ice column from the sample surface using the lateral pushing method.

### De‐icing and Anti‐icing Experimental Setup

Figure S3 (Supporting Information) is the schematic of the de‐icing and anti‐icing experimental setup. The MRS is placed on a semiconductor cooler attached to a cooling plate. Constant temperature circulation is connected to the cooling plate to provide a low temperature condition. A capillary tube is positioned above the surface, which connects to a microliter syringe via a flexible tubing. Besides, a perspex shell with dimensions of 100 mm in length and width and 200 mm in height is used to cover the surface during the experiment. Magnet blocks are placed with a controlled stand frame to change the height of the magnet. Prior to the experiment, silica gel desiccant beads are placed inside the shell to reduce humidity (below 10%). Type T thermocouples (KAIPUSEN, T‐0.08) are placed in the PDMS layer of the MRS to test the environment, which, as well as the surface temperature, is acquired by a data acquisition unit (Agilent instruments 34972A). The surface temperature is controlled by a semiconductor cooler through adjusting the DC power supply. Deionized droplets are generated by a microliter syringe and a needle. The freezing and the de‐icing process is captured with a high‐speed camera (Phantom V2012) after the injected droplet falls onto the MRS and totally freezes. During the de‐icing process, the magnet block is vertically lifted from the level of the surface to change the magnetic field magnetization. In addition, a cold light (Nanya special lighting factory, XD‐300) is adopted to backlight the ice particles on the surface.

## Conflict of Interest

The authors declare no conflict of interest.

## Supporting information



Supporting Information

## Data Availability

The data that support the findings of this study are available from the corresponding author upon reasonable request.
