# Supplementary material for: Micro‐Scale Ice Shoveling Effect Induced by Magnetic‐Responsive Microfins
Source: Adv Sci (Weinh). 2024 Oct 23;11(46):2408594. doi: 10.1002/advs.202408594 (PMC11633509; doi:10.1002/advs.202408594)
Supplement: Supplementary file 1 — Supporting Information [file ADVS-11-2408594-s001.docx]

Supplementary Materials for

**Micro-scale ice shoveling effect induced by magnetic-responsive microfins**

Yiyi Chen *et al.*

*Corresponding author. Email: xmao@bit.edu.cn

**This PDF file includes:**

Supplementary Text

Figs. S1 to S3

Supplementary Text

Figure S1. shows the top view of the ice base on fin 1 and fin 2.. $A_{1}$and $A_{2}$ are the contact areas between the ice and fin 1 and fin 2, respectively. $A_{1}$ is given as,

| $A_{1}=2L_{2}T_{fin}$ | (S1) |
| --- | --- |
| $L_{2}=\left[ R_{b}^{2}-\left( {L_{1}-R}_{b} \right)^{2} \right]^{0.5}$ | (S2) |

where $R_{b}$ is the radius of the ice base, $T_{fin}$ is the thickness of the fin, $L_{1}$ is the distance from the top of fin 1 to the end of the ice base on fin 2, and $L_{2}$ is one half of the contact length on the top of fin 1. $A_{2}$ is expressed as,

| $A_{2}=\pi R_{b}^{2}-\left[ \frac{arcsin\left( \frac{L_{2}}{R_{b}} \right)}{180}\pi R_{b}^{2}-L_{2}\left( L_{1}-R_{b} \right) \right]$ | (S3) |
| --- | --- |


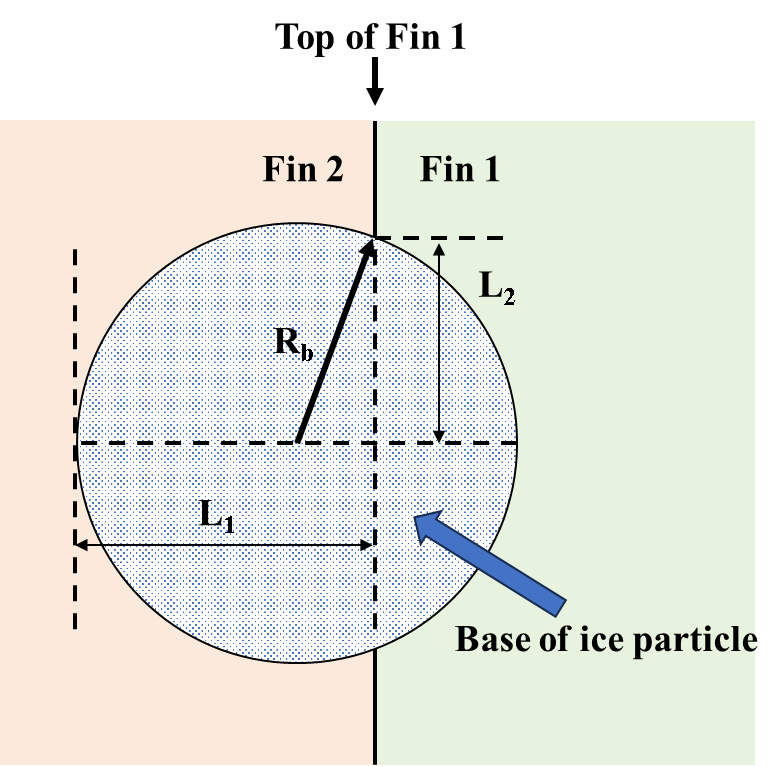


**Figure S1.** Schematic illustration of top view of the ice base on fin 1 and fin 2


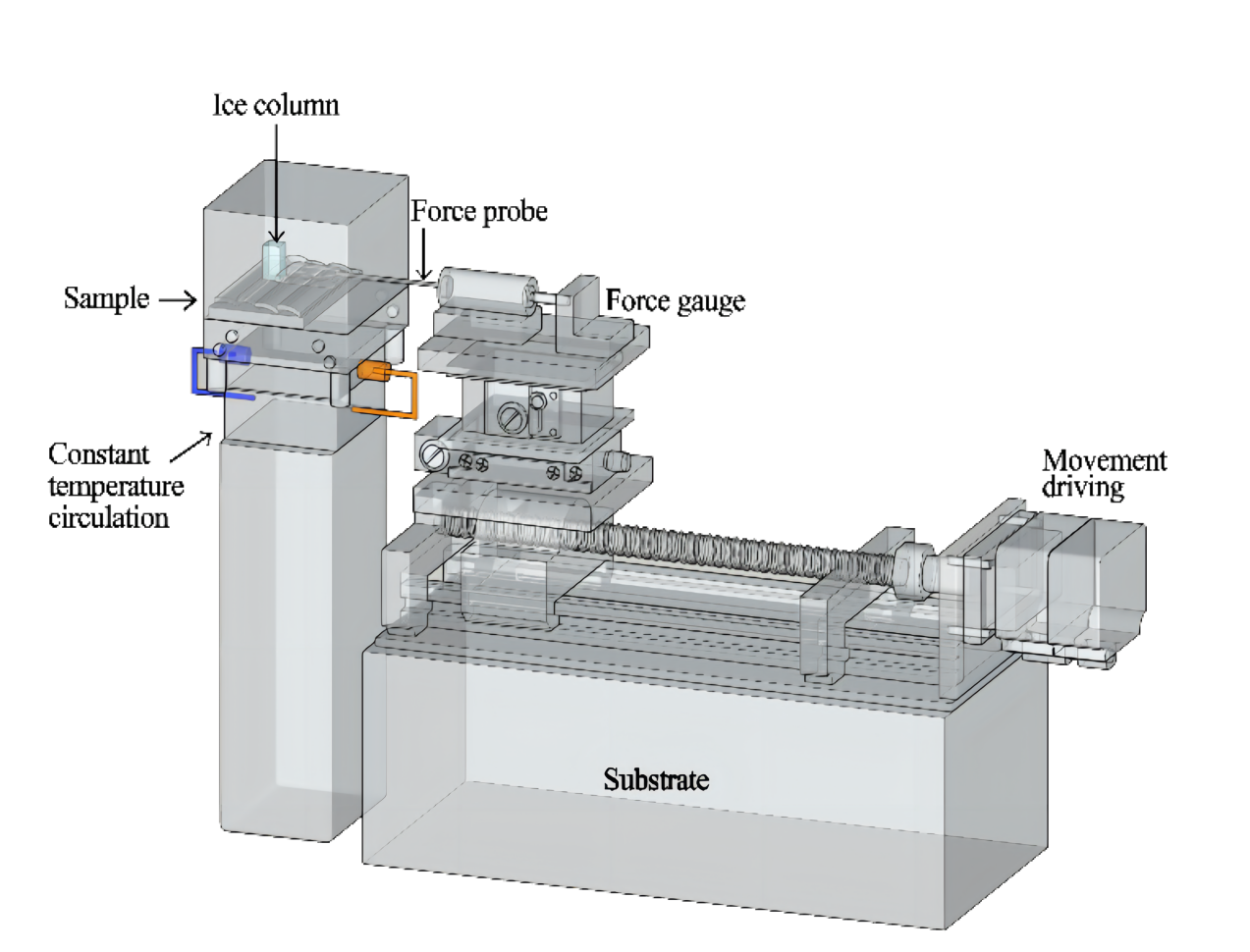


**Figure S2.** Experimental setup for measurement of the shear ice adhesion force.


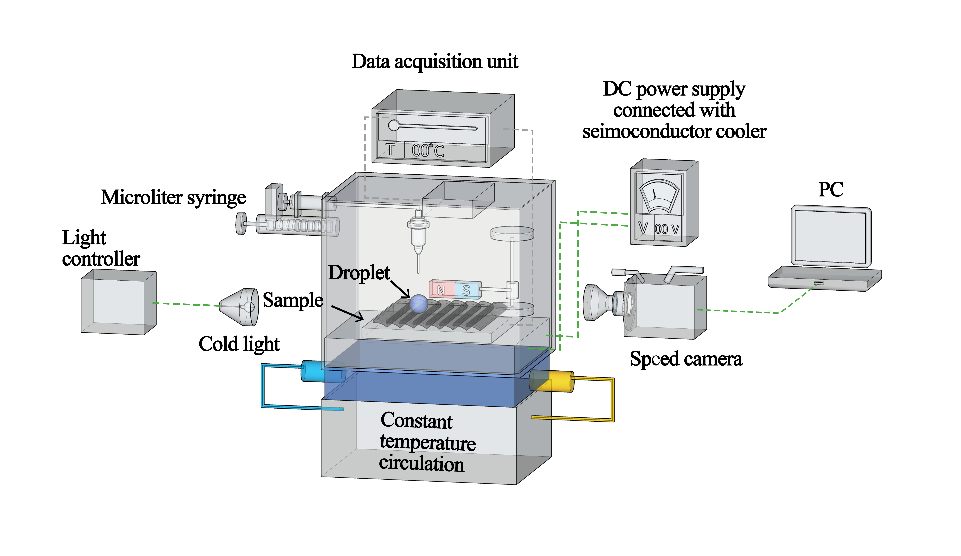
**Figure S3.** Schematic of de-icing experiment setup.
